# Ecological risk assessment and spatial–temporal differentiation of soil and water resources in the Hefei metropolitan area

**DOI:** 10.1038/s41598-024-59246-6

**Published:** 2024-04-11

**Authors:** Shuhang Zhao, Gang He, Jie Li, Xia Yang, Xiaoyu Hou, Ting Wu, Shangyun Zhang

**Affiliations:** 1 School of Economics and Management, Anhui University of Science and Technology, Huainan, 232001 Anhui China; 2 School of Mathematics and Big Data, Anhui University of Science and Technology, Huainan, 232001 Anhui China

**Keywords:** Soil and water resources, Ecological risk, CRITIC, TOPSIS, Kernel density, Spatial markov chain, Ecology, Environmental social sciences, Natural hazards

## Abstract

There are important ways to solve the ecological risk problems of regional water resources and soil resources, and to promote the benign development of soil and water resources, involving scientific evaluation of the ecological risk of soil and water resources in Hefei metropolitan area, clarifying the intrinsic evolution law of ecological risk and identifying the characteristics of spatial and temporal variations. Based on the conceptual model of “ST-QS-RR”, the evaluation indicator system is constructed, the CRITIC method is used to assign weights, and the TOPSIS method, kernel density method, markov chain and resistance model are used to measure and analyse the spatial and temporal characteristics of ecological risk of soil and water resources, and to explore the main factors that cause ecological risk of soil and water resources. The results of the study show that: (1) Hefei metropolitan area and its cities show a steady decline and the characteristics of “high in the north and low in the south, high in the west and low in the east”. (2) Most of the subsystems in the Hefei metropolitan area and the cities show a decreasing trend, with its resistance factors mainly concentrated in the QS system. (3) There is club convergence in Hefei metropolitan area. When the type of adjacent domain is higher, the change of risk type is more sensitive.

## Introduction

Soil is the foundation of all things, water is the source of life, and soil and water resources are the ecological basis for human survival and development. With a series of ecological risk problems such as soil erosion^1^, water quality deterioration^2^, land desertification^3^ and so on, the root cause is from the unreasonable development and utilization of soil and water resources. The ecological risk of soil and water resources has seriously threatened the soil and water conservation and ecological civilization construction of cities. The transformation problem of resource-based cities needs to be solved urgently. Therefore, the ecological risk assessment of cities is imminent^4^.

Risk evaluation was originally used to calculate the probability of winning, and later flourished in the seventeenth century in England for pensions and insurance premiums and in the Netherlands for the probability of safety of ship navigation^5,6^. Risk evaluation is the product of a compromise between science and law, and as Bernstein suggests, “the scientific method of calculating uncertainty^5^”, risk evaluation has the universal characteristics of decision making and uncertainty of outcome. Nowadays, risk assessment is not only used in the financial and insurance industries^7^, but also in various fields such as engineering projects^8^, healthcare^9^ and ecological environment^10^.

Ecologists have proposed the term “ecological risk”, which specifically refers to the risk to non-human ecosystems, as opposed to “environmental risk”, which refers to the risk to humanity^11^. With the continuous advancement of green urbanisation, socie-economy need to constantly coordinate the relationship between soil and water resources demand and allocation^12^. Water resources risks are generally assessed in terms of water quality^13^ and water quantity^14^, and soil resource risks are generally assessed in terms of land use^15^ and land pollution^16^. Soil and Water resources restrict each other and complement each other.

The importance and attention of domestic and foreign scholars to soil and water ecological risks are constantly increasing. Combining environment, economy and society aspects, Liao et al. constructed the coupled ecosystem risk evaluation indicator system of soil and water resources, and used the improved entropy weighting method and weighted summation method for evaluation^17^. Wu et al. selected the index related to the effect of soil and water use, constructed the ecological risk assessment and countermeasure system of soil and water use, and analysed and researched the dynamics and spatial changes of soil and water ecological risk^18^. Yu et al. used the Malmquist DEA model and the coupled coordination model to construct an agricultural ecological risk assessment system that considers the coupling of soiland water resources^19^. Spatial correlation analysis was conducted based on the degree of risk related WLR. Mugambiwa et al. explored climate governance based on Indigenous Knowledge Systems (IKS) in soil and water resource management, using a qualitative multi case study exploratory design^20^. Iori et al. used comparative maps to compare the impacts of land use on soil quality in Brazilian riparian areas^21^. Cultivated areas adjoining a watercourse can lead to severe changes in soil properties.

Although academic research on ecological risk evaluation is fruitful, there are little research results specifically for soil and water resources. The deficiencies in the research are reflected in these aspects: first, the evaluation indicator system is not systematically constructed from the perspective of the risk sources and risk points existing in soil and water resources. Second, the selected research method fails to evaluate the research object in multiple dimensions and identify the resistance factors. Third, the method of assignment is dominated by the entropy weight method, which fails to eliminate the correlation of evaluation indicators.

Xu et al. selected 28 evaluation indicators based on the DPSIR model, only 2 of which were directly related to ecological risks (soil erosion rate and water pollution index), and used the coefficient of variation to weight the indicators^22^. Jiang et al. selected 14 evaluation indicators from the perspectives of supply and demand, only 1 of which was directly related to ecological risks (fertilizer application rate per unit of arable land), and used the entropy weight method to weight the indicators^23^. Liao et al. selected 34 evaluation indicators involving the three aspects of environment, economy, and society, only 2 of which were directly related to ecological risks (frequency of drought and flood disasters, pesticide application rate), and used the entropy weight method to weight the indicators^17^. These studies did not identify resistance factors, and the weighting method did not eliminate the correlation between indicators.

This article makes corresponding improvements to the above shortcomings, carefully screening indicators from the perspectives of soil and water resources, and from the three dimensions of "threat state regulation". Secondly, it conduct resistance diagnosis on the ecological risks of soil and water resources in various cities, and identify the main resistance factors in each city. Finally, by using the CIRTIC method to weight indicators, the correlation between indicators can be calculated, effectively reducing the impact of excessive correlation between relevant indicators on the evaluation results.

To sum up, there is no research specifically combining water resources and soil resources in Hefei metropolitan area, and the indicator system for quantitative research in Hefei metropolitan area needs to be improved. In this paper, considering the complexity and uncertainty of ecological risk of soil and water resources^24^, we respectively explore the three aspects of security threat, quality status and risk regulation at the guideline layer, and combine the socio-economic and resource environment factors to carry out multi-dimensional exploration. With the help of CRITIC method to assign weights to the evaluation indicators, TOPSIS method was used to evaluate the ecological risk level of soil and water resources in Hefei metropolitan area from 2012 to 2021, and kernel density method and markov chain were combined to identify the characteristics of spatio-temporal differentiation. Finally, the resistance model is applied for resistance diagnosis. The results of the study can truly reflect the ecological risk status of soil and water resources in Hefei metropolitan area in recent years, and provide theoretical support for effectively reducing the ecological risk level of soil and water resources.

## Materials

### Overview of the study area

The metropolitan area was first proposed by Japan in 1960 and evolved from the “metropolitan region” proposed by the United States in 1910^25^. The Hefei metropolitan area is a functional area centred on Hefei City and radiating to the seven surrounding cities of Bengbu City, Huainan City, Chuzhou City, Wuhu City, Tongcheng City (a county-level city) in Anqing City, Lu'an City and Ma'anshan City^26^.

**Figure 1:**
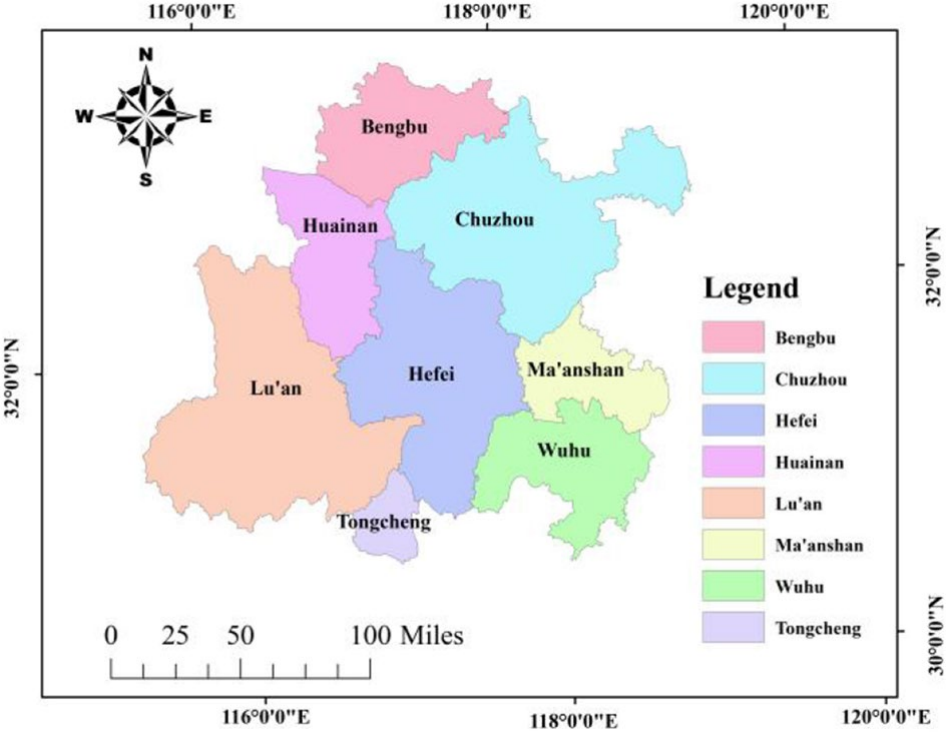
Map of the administrative districts.

As can be seen from Fig. 1, the area as of Lu’an City is the largest, followed by Chuzhou City. The prefecture-level city with the smallest area is Ma’anshan. Except for Bengbu City, several other cities border Hefei City. Prefecture-level cities and county-level cities have different spatial scales, so Tongcheng is not included in the study.

**Figure 2:**
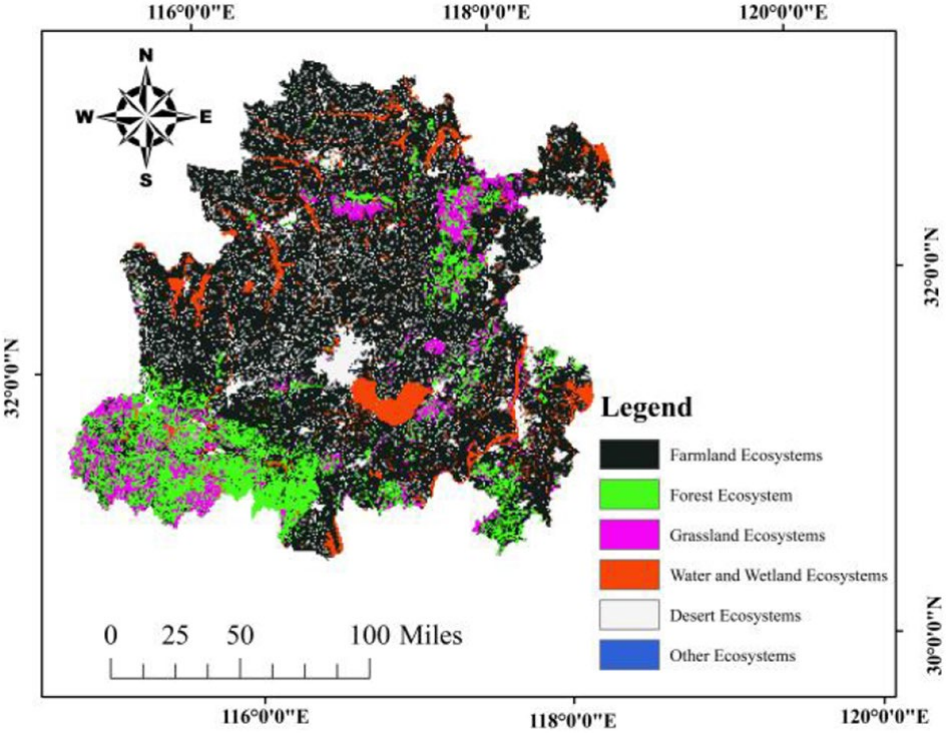
Spatial distribution of terrestrial ecosystems.

Figure 2 is based on 1 km raster data at a spatial resolution of 1:100,000 scale. The terrestrial ecosystems in the Hefei metropolitan area can be divided into six types. Among them, farmland ecosystems have the largest area, followed by forest ecosystems, and other ecosystems have the smallest area.

**Figure 3:**
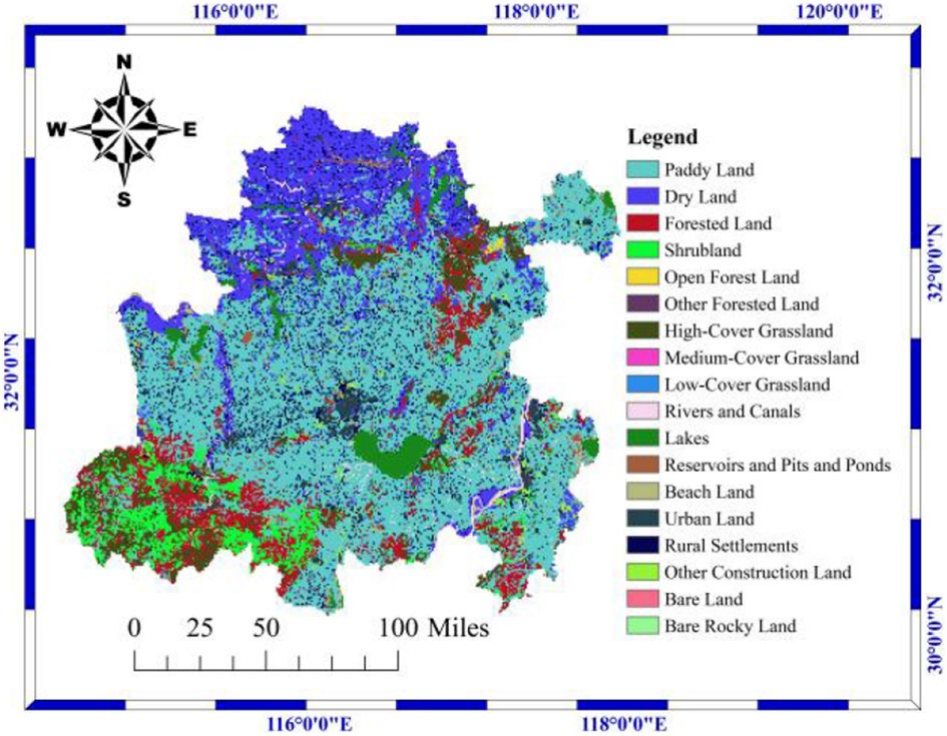
Map of land-use remote sensing monitoring data.

Figure 3 is based on 1-km raster data from the China Multi-Period Land Use Remote Sensing Monitoring Dataset. The Hefei metropolitan area can be divided into 18 types according to land resources and their utilization attributes. Paddy land has the largest area, followed by dry land, and the smallest area is bare rocky land.

**Figure 4:**
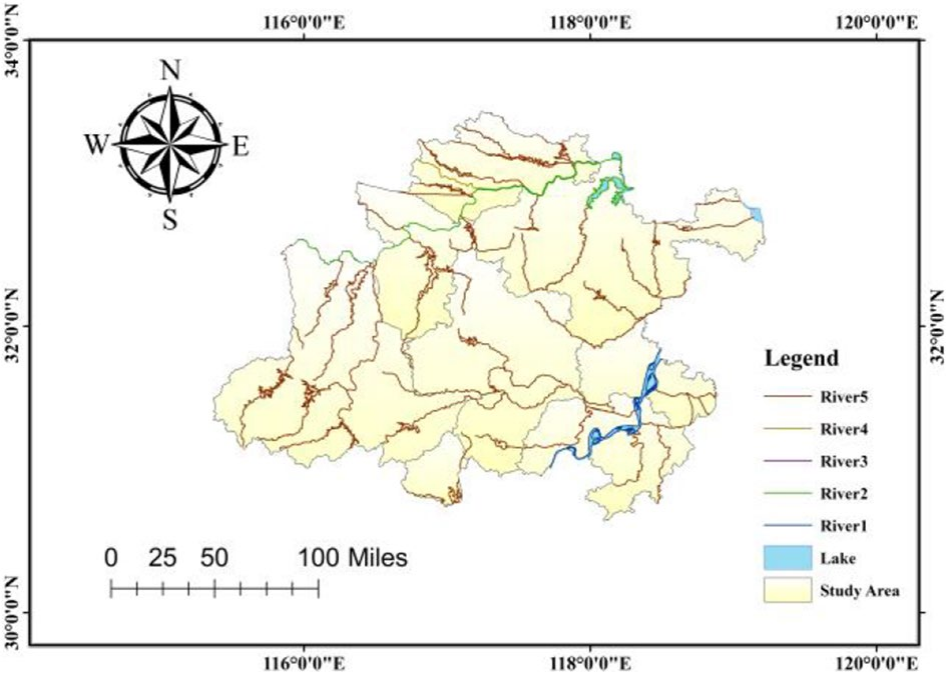
Water system map.

Figure 4 is based on elemental data of Class 1–5 rivers and major lakes in China. The Hefei metropolitan area can be divided into first-class rivers (Yangtze River), second-class rivers (Huaihe River), fourth-class rivers (Eddy River), fifth-class rivers (Tuo River, Beifei River, Baita River, etc.) and major lakes (Hongze Lake, Gaoyou Lake, etc.) based on the characteristics of the water system.

**Figure 5:**
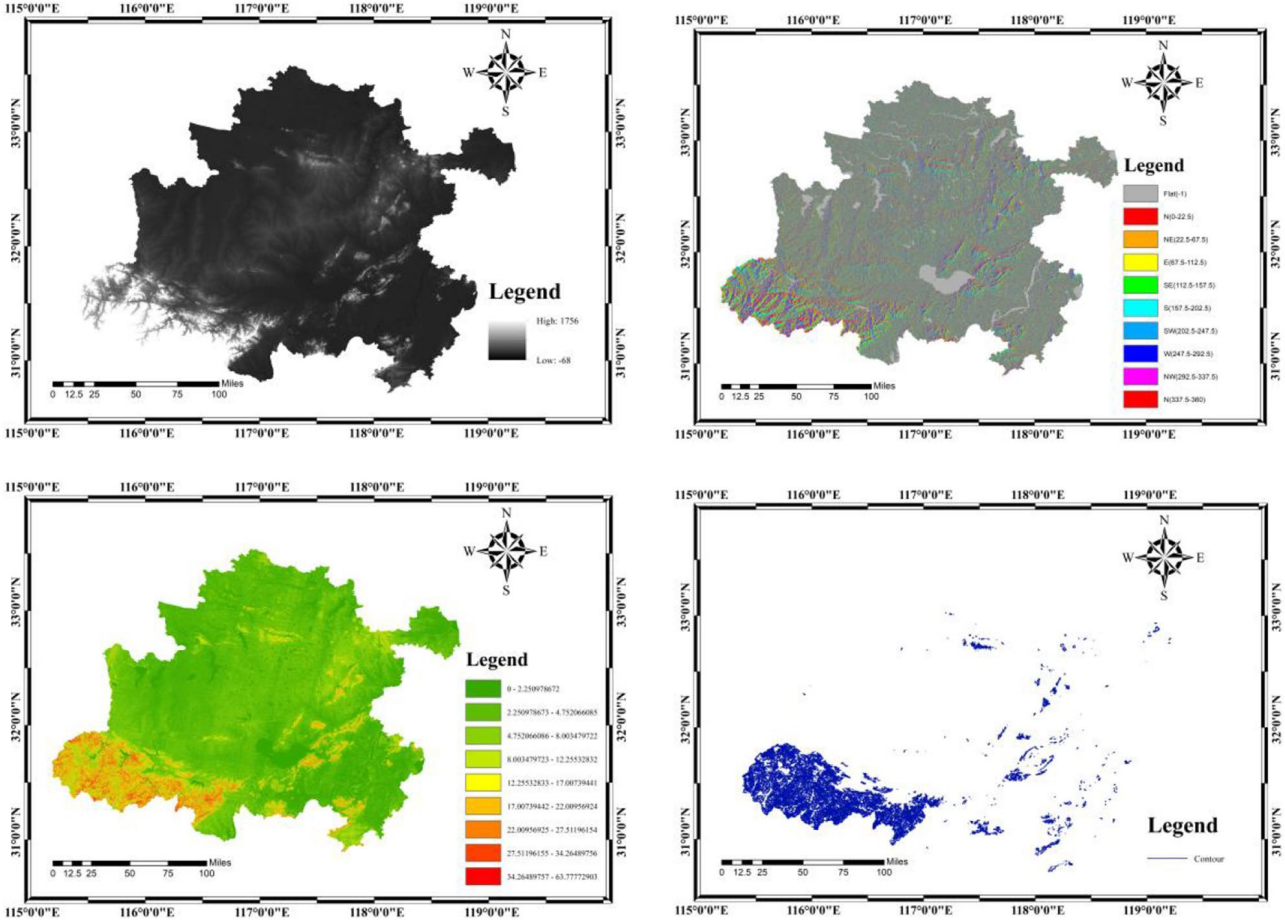
Map of DEM 30 M data. (height, slope direction, slope gradient and contour).

Figure 5 is the completion of the GDEMV3 30 M resolution digital elevation data plotting based on the Geospatial Data Cloud. Hefei metropolitan area observes the height according to the DEM, the maximum value is 1756 m, the minimum value is − 68 m. Hefei metropolitan area outputs the data of slope direction, slope gradient and contour according to the DEM, and the specific distribution can be seen very intuitively in Fig. 5.

As can be seen from Table 1, the soil and water erosion area of Hefei metropolitan area has undergone continuous changes from 2021 to 2022.

**Table 1:** Dynamics of soil and water erosion area in 2022.

City	Soil and water erosion area (km^2^)	Soil and water conservation rate (%)	Change of soil and water erosion area (km^2^)
Hefei	610.98	94.69	− 15.34
Bengbu	43.21	99.27	− 2.19
Huainan	40.11	99.29	− 0.82
Chuzhou	981.1	92.68	− 3.97
Lu'an	2137.67	86.17	− 34.69
Ma'anshan	209	94.83	1.19
Wuhu	207.78	96.51	− 6.38

In terms of soil and water erosion area, Lu'an City is the largest, reaching 2,137.67 km^2^, followed by Hefei City, which has an area of 610.98 km^2^, and the smallest is Huainan City, which has only 40.11 km^2^. In terms of soil and water conservation rate, Huainan and Bengbu City are the best, and the two cities are basically equal both above 99%, only Lu'an City is lower than 90%. In terms of the change of soil and water erosion area, Lu'an City has the biggest change, followed by Hefei City, and the smallest change is in Huainan City.

### Data sources

The data for this study come from *the Anhui Statistical Yearbook*^27^, *the Anhui Water Resources Bulletin*^28^, *the Anhui Soil and Water Conservation Bulletin*^29^, the Resource and Environmental Science Data Centre of the Institute of Geographic Sciences and Resources of the Chinese Academy of Sciences, the Geospatial Data Cloud, and the AliCloud Data Visualisation Platform, and some of the evaluation indicator values are calculated based on formula.

### System of evaluation indicators

Combining the previous research results^19,30–33^, considering the actual situation of Hefei metropolitan area, and combining the principles of accessibility and scientificity of ecological evaluation indicators, 15 representative evaluation indicators are selected to construct the evaluation indicator system from the three guideline layers of security threat, quality status and risk regulation, and integrating the two major factors of socio-economics and resources environment, as shown in Table 2.

**Table 2:** Evaluation indicator system.

Guideline	Factor	Indicator	Significance
Security threat	Socio-economic	I_1_—Per capita water use for urban public services	Degree of access to water for social security
		I_2_—Industrial wastewater discharge of 10,000 yuan GDP	Industrial emissions from economic development
		I_3_—Urban population density	Population distribution in urban development
	Resource environment	I_4_—Proportion of groundwater extraction	Degree of exploitation of water resources
		I_5_—Proportion of land resettlement	Land use of agricultural cropland
Quality status	Socio-economic	I_6_—Urbanisation rate	Degree of socio-demographic urbanisation
		I_7_—Green space in parks per capita	Per capita level of park green space
		I_8_—Proportion of green squares to construction land	The area of Greenland Square
	Resource environment	I_9_—Matching coefficient of agricultural water and soil resources	Degree of match between agricultural water and soil
		I_10_—Per land rainfall	Precipitation capacity per unit area
		I_11_—Water resources per capita	Per capita possession of water resources
Risk regulation	Socio-economic	I_12_—Proportion of public budget expenditure on agriculture, forestry and water	The importance of basic industries
		I_13_—Per capita expenditure on research	Intensity of investment in scientific research funds
	Resource environment	I_14_—Eco-environmental water supplement ratio	Water compensation degree of ecological environment
		I_15_—Industrial Wastewater Treatment Rate	Efficiency of industrial wastewater treatment

### Classification criteria

The criteria of ecological risk grade of soil and water resources directly affect the accuracy of the results, this paper evaluates the ecological risk level of soil and water resources in Hefei metropolitan area by dividing the risk value into five ecological risk grades (see Table 3 for details) according to the research results of Liao et al.^17^, Wu et al.^18^ and Yu et al.^19^.

**Table 3:** Ecological risk grade criteria for soil and water resources.

Risk value	0–0.25	0.25–0.50	0.50–0.70	0.70–0.85	0.85–1.00
Grade symbol	I	II	III	IV	V
Risk grade	Extremely low	Lower	General	Higher	Extremely high
Warning grade	No	Light	Medium	Heavy	Huge

## Methods

### CRITIC method

Diakoulaki et al.^34^ proposed the CRITIC (Criteria Importance Through Intercrieria Correlation) method, which comprehensively measures the comparative strength and conflicting characteristics of the evaluation indicators.The CRITIC method, PCA (Principal Component Analysis) and GRA (Grey Correlation Analysis) and other objective assignment methods can well avoid the errors caused by subjectivity, and the CRITIC method optimally handles the degree of correlation between evaluation indicators, which is more scientific and reasonable than other methods, and is now applied to various types of ecological risk evaluation^30,35,36^.

Assuming that there are *m* evaluation objects and *n* evaluation indicators, then *a*_*ij*_ denotes the *j*th evaluation indicator of the *i*th evaluation object, and the specific steps are shown below:

1. Data were standardised using the max–min method, with formula (1) representing positive indicators and formula (2) representing negative indicators.
  1 $$x_{ij} = \frac{{aij - \min \left\{ {aij} \right\}}}{{\max \{ aij\} - \min \left\{ {aij} \right\}}}\;(i = 1,2, \ldots ,m,j = 1,2, \ldots ,n)$$
  2 $$x_{ij} = \frac{{\max \{ aij\} - aij}}{{\max \{ aij\} - \min \left\{ {aij} \right\}}}\;(i = 1,2, \ldots ,m,j = 1,2, \ldots ,n)$$
2. The mean is calculated from formula (3), the standard deviation is derived from formula (4), and the variability of the evaluation indicators is expressed by the standard deviation.
  3 $$\overline{x}_{j} = \frac{1}{m}\sum\limits_{i = 1}^{m} {x_{ij} }$$
  4 $$S_{j} = \sqrt {\frac{{\sum\limits_{i = 1}^{m} {(x_{ij} - \overline{x}_{j} )^{2} } }}{m - 1}}$$
3. The correlation coefficient is derived using formula (5), and the conflictiveness of the evaluation indicators is derived from formula (6):
  5 $$r_{{jj{\prime} }} = \rho (X_{j} ,X_{{j{\prime} }} ) = \frac{{{\text{cov}} (X_{j} ,X_{{j{\prime} }} )}}{{\sigma_{{X_{j} }} \sigma_{{X_{{j{\prime} }} }} }} = \frac{{E(X_{j} X_{{j{\prime} }} ) - E(X_{j} )E(X_{{j{\prime} }} )}}{{\sqrt {E(X_{j}^{2} ) - E^{2} (X_{j} )} \sqrt {E(X_{{j{\prime} }}^{2} ) - E^{2} (X_{{j{\prime} }} )} }}$$
  6 $$R_{j} = \sum\limits_{j = 1}^{n} {(1 - r_{{jj{\prime} }} )}$$
4. The information amount is calculated by combining variability and conflictiveness with the help of formula (7):
  7 $$C_{j} = S_{j} \times R_{j}$$
5. The weights of the evaluation indicators are calculated with the help of the information amount by formula (8):
  8 $$W_{j} = \frac{{C_{j} }}{{\sum\limits_{j = 1}^{n} {C_{j} } }}$$

### TOPSIS method

The TOPSIS method^37^ is a near-ideal solution evaluation method proposed by Yoon in 1980. The closeness of each evaluation indicator to the optimal programme is derived by normalising the homotrended data to the optimal and worst programmes, assigning weights using the CRITIC method, and calculating the distance of each evaluation indicator to the optimal and worst programmes, as follows:

1. The data were homotrended, and the low merit indicators were transformed using the inverse method to obtain *B* = {*b*_*ij*_}_*m*×*n*_. The data after the homotrending process were normalised as shown in formula (9):
  9 $$y_{ij} = \frac{{b_{ij} }}{{\sqrt {\sum\limits_{i = 1}^{m} {b_{ij}^{2} } } }}$$
2. Calculate the positive and negative ideal solutions *Y*^+^ and *Y*^*−*^ for each evaluation indicator:
  10 $$\begin{gathered} Y^{ + } = \left\{ {\max y_{ij} |j = 1,2, \ldots ,n} \right\} = \left\{ {y_{1}^{ + } ,y_{2}^{ + } , \ldots ,y_{n}^{ + } } \right\} \hfill \\ Y^{ - } = \left\{ {\min y_{ij} |j = 1,2, \ldots ,n} \right\} = \left\{ {y_{1}^{ - } ,y_{2}^{ - } , \ldots ,y_{n}^{ - } } \right\} \hfill \\ \end{gathered}$$
3. Calculate the distance of each evaluation indicator from the optimal and worst programme *D*^+^ and *D*^*−*^:
  11 $$\begin{gathered} D_{j}^{ + } = \sqrt {\sum\limits_{j = 1}^{n} {W_{j} (y_{j}^{ + } - y_{ij} )^{2} } } \hfill \\ D_{j}^{ - } = \sqrt {\sum\limits_{j = 1}^{n} {W_{j} (y_{j}^{ - } - y_{ij} )^{2} } } \hfill \\ \end{gathered}$$
4. The proximity of the *ith* evaluation indicator to the optimal programme (closeness) *T*_*j*_:
  12 $$T_{j} = \frac{{D_{j}^{ - } }}{{D_{j}^{ + } + D_{j}^{ - } }}$$

The value range of *T*_*j*_ is [0, 1], the closer to 1, means the more tend to the optimal level, and vice versa, the more connected to 0, means the more tend to the worst level. The *T*_*j*_ represents the ecological safety value of soil and water resources.

(5) The sum of the ecological safety value of soil and water resources and the ecological risk value of soil and water resources is 1^15^, and the formula is shown below:

13 $$U_{j} = 1 - T_{j}$$

The ecological risk value of soil and water resources *U*_*j*_ is in the range of [0, 1], the closer to 1, the higher the risk level and the higher the warning level, and vice versa, the closer to 0, the lower the risk level and the lower the warning level.

### Kernel density estimation

Kernel density estimation^38^ can be used to analyse the distribution pattern of ecological risk of soil and water resources. In this paper, Gaussian kernel function is chosen to do two-dimensional kernel density map, and three-dimensional kernel density map. With the help of both of them, the distribution dynamics and evolution trend of ecological risk of soil and water resources in Hefei metropolitan area are estimated. The formula is shown below:

14 $$f\left( x \right) = \frac{1}{Nh}\sum\limits_{i = 1}^{N} {K\left( {\frac{{x - X_{i} }}{h}} \right)}$$

where *N* is the number of observations, *h* is the bandwidth, *K* is the kernel function, *X*_i_ is the closeness value of each city in Hefei metropolitan area, and *x* is the mean value. Kernel density estimation is more sensitive to the bandwidth *h*. The larger *h is*, the lower the accuracy of the kernel density function, and the smoother the curve is. On the contrary, the smaller *h* is, the higher the accuracy of the kernel density function is, and the more obvious the curve angles are. Therefore, a smaller value of *h is* chosen for the kernel density function curve while ensuring a beautiful curve.

### Markov chain

Markov chain can reflect the dynamic development characteristics of the research object, and the dynamic evolution process is analysed by introducing the transfer probability matrix^32,39^, which reflects the upward or downward mobility of the research object. In this paper, based on the principle of Markov chain, it is used to explore the transfer probability of the ecological risk grade of soil and water resources in Hefei metropolitan area over time. The ecological risk values of soil and water resources were classified into four types according to quartiles, which were low risk L (< 0.7180), medium–low risk ML (0.7180–0.7510), medium–high risk MH (0.7510–0.8100), and high risk H (> 0.8100), and the matrix was shown in Table 4.

**Table 4:** Markov transfer probability matrix.

*t*\*t* + 1	L	ML	MH	H
L	*P*_11_	*P*_12_	*P*_13_	*P*_14_
ML	*P*_21_	*P*_22_	*P*_23_	*P*_24_
MH	*P*_31_	*P*_32_	*P*_33_	*P*_34_

The *P*_*ij*_ in the matrix represents the probability that the ecological risk grade of municipal soil and water resources is of *i*th type at the moment *t* and of *j*th type at the moment *t* + *1*. The formula is shown below:

15 $$P_{ij} = \frac{{z_{ij} }}{{z_{i} }}$$

where *z*_*ij*_ is the sum of the number of cities with ecological risk grade of soil and water resources of *i*th type at moment *t* that will be shifted to *j*th type at the next moment; and *z*_*i*_ is the sum of the number of cities with ecological risk grade of soil and water resources of *i*th type. If the ecological risk grade of soil and water resources remains unchanged, it means that the ecological risk of soil and water resources in the city remains stable; if the ecological risk grade of soil and water resources increases, it means that the ecological risk grade of soil and water resources in the city is shifted upwards; on the contrary, the ecological risk grade of soil and water resources is shifted downwards.

In the traditional Markov chain mentioned above, the transfer probability is unaffected by the surrounding things and does not take the influence between things into account. However, with the deepening of research, scholars began to pay attention to the influence of geographical location on the transfer probability. Considering the spatial spillover effect of ecological risk of soil and water resources, the concept of "spatial lag" is introduced, and the ecological risk value of soil and water resources in neighbouring cities is used to classify the lag grade, and the matrix is shown in Table 5.

**Table 5:** Spatial Markov chain transfer probability matrix. (K = 4).

*Lag*	*t*\*t* + 1	L	ML	MH	H	*Lag*	*t*\*t* + 1	L	ML	MH	H
1	L	*P*_11|1_	*P*_12|1_	*P*_13|1_	*P*_14|1_	3	L	*P*_11|3_	*P*_12|3_	*P*_13|3_	*P*_14|3_
	ML	*P*_21|1_	*P*_22|1_	*P*_23|1_	*P*_24|1_		ML	*P*_21|3_	*P*_22|3_	*P*_23|3_	*P*_24|3_
	MH	*P*_31|1_	*P*_32|1_	*P*_33|1_	*P*_34|1_		MH	*P*_31|3_	*P*_32|3_	*P*_33|3_	*P*_34|3_
	H	*P*_41|1_	*P*_42|1_	*P*_43|1_	*P*_44|1_		H	*P*_41|3_	*P*_42|3_	*P*_43|3_	*P*_44|3_
2	L	*P*_11|2_	*P*_12|2_	*P*_13|2_	*P*_14|2_	4	L	*P*_11|4_	*P*_12|4_	*P*_13|4_	*P*_14|4_
	ML	*P*_21|2_	*P*_22|2_	*P*_23|2_	*P*_24|2_		ML	*P*_21|4_	*P*_22|4_	*P*_234_	*P*_24|4_
	MH	*P*_31|2_	*P*_32|2_	*P*_33|2_	*P*_34|2_		MH	*P*_31|4_	*P*_32|4_	*P*_33|4_	*P*_34|4_
	H	*P*_41|2_	*P*_42|2_	*P*_43|2_	*P*_44|2_		H	*P*_41|4_	*P*_42|4_	*P*_43|4_	*P*_44|4_

*P*_*ki|j*_ in the matrix denotes the probability that the ecological risk grade of soil and water resources of class *k* will shift from *i* to *j* at the next moment. The spatial lag value is calculated as:

16 $$Lag_{a} = \sum\limits_{b = 1}^{n} {W_{ab} } Y_{b}$$

where *Y*_*b*_ is the observed value of city *b*, *Lag*_*a*_ is the spatial lag value of city *a*, *n* is the total number of cities, and the spatial weight matrix *W*_*ab*_ denotes the spatial neighbourhood of city *a* and city *b*. In this paper, the spatial relationship is defined using the adjacency principle, i.e., the area adjacency value is 1, otherwise it is 0.

### Resistance model

In order to guarantee the rational planning and development of soil and water resources, and to formulate and adjust the comprehensive management policy, this study applies the resistance model^40^ to analyse and diagnose the main indicators affecting the ecological risk of soil and water resources. The specific formula is as follows:

17 $$O_{i} = \frac{{S_{i} W_{i} }}{{\sum\limits_{i = 1}^{n} {\left( {S_{i} W_{i} } \right)} }}$$

where the resistance value *O*_*i*_ indicates the degree of influence of the indicator on the ecological risk of soil and water resources; the bias of the indicator *S*_*i*_ = 1*-X*_*ij*_, indicates the difference between the evaluation indicator and the optimal value; the contribution of the factor *W*_*i*_ (weight), indicates the size of the contribution of the evaluation indicator to the ecological risk of soil and water resources.

## Results

### Time series analysis

**Figure 6:**
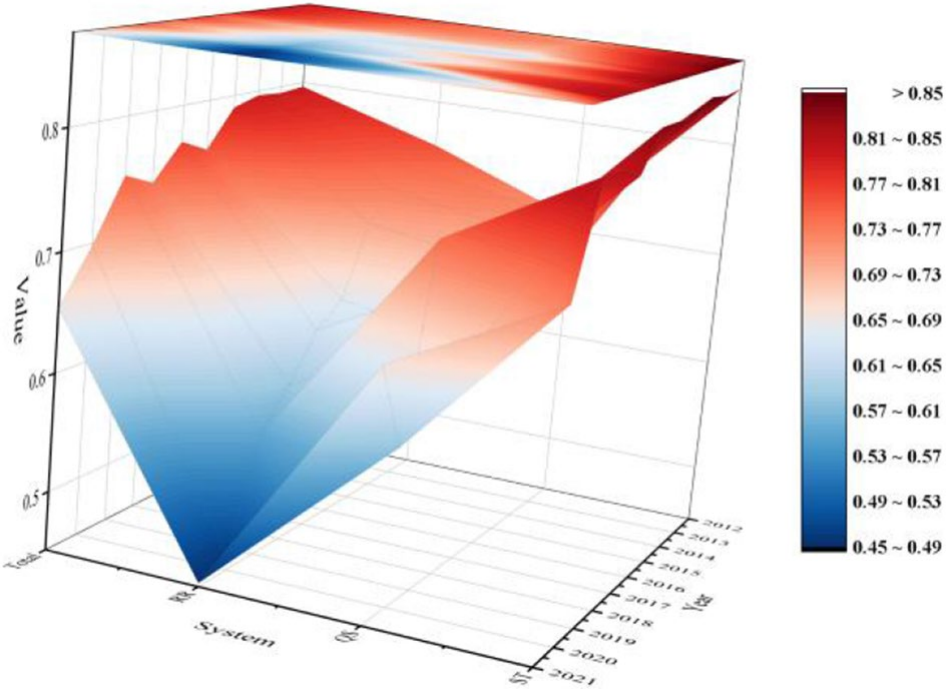
3D surface map of ecological risk in soil and water resources.

The risk values of ecological risk of soil and water resources of Total system and of each subsystem in the Hefei metropolitan area were calculated using the TOPSIS method from 2012 to 2021, as shown in Fig. 6.

The ecological risk level of soil and water resources in the Hefei metropolitan area was basically flat from 2012 to 2014, with the risk grade remaining at higher and the warning grade being heavy. The ecological risk level of the ST system was basically flat, remaining around 0.85, with the risk grade remaining at extremely high and the warning grade being huge in 2012 and 2014, but the risk grade fell to higher and the warning grade being heavy in 2013. The QS system went up and then down, and the RR system showed a decreasing trend, with the risk grade remaining at higher and the warning grade being heavy in all cases.

The ecological risk of soil and water resources in the Hefei metropolitan area from 2015 to 2019 shows a “W”-shaped trend, with the risk grade being higher and the warning grade being heavy. The ecological risk level of the ST system in the first 3 years is basically the same and stays around 0.84, but slightly decreases in the last 2 years, with the risk grade being higher and the warning grade being heavy. The change trend of QS system is consistent with that of the overall system, showing a “W” shape. In the first 4 years, the risk grade was general and the warning grade was medium, and in 2019, the risk grade was raised to higher and the warning grade was heavy. The ecological risk level of the RR system has continued to decline in the past 5 years, with the risk grade dropping from higher to general and the warning grade dropping from heavy to medium.

The ecological risk of soil and water resources in the Hefei metropolitan area in 2020–2021 shows a decreasing trend, falling below 0.7 in both years, with the risk grade at general and the warning grade at medium. Both the ST system and the RR system show a decreasing trend, and the QS system shows an increasing trend. The ST system shows a larger decrease, amounting to 10.43%, but the risk grade still remains at higher and the warning grade at heavy. The QS system shows a larger increase, amounting to 13.23%, with the risk grade still remaining at general and the warning grade at medium. The RR system showed a smaller decrease of 1.95%, with the risk grade at lower and the warning grade at light.

Overall, the trend of ecological risks in water and soil resources in the Hefei metropolitan area is relatively stable, showing a steady downward trend. The Hefei metropolitan area shows a trend of “flat-down-up-down-up-down”. The risk level is at a higher risk in the early stage, and it drops to a general risk in the later stage, and finally tends to be a general risk. From the subsystem perspective, the ST system shows a trend of “flat-down-flat-down-up-flat-down”, and the risk level has been at a higher risk, with a tendency to return to a general risk. The QS system shows a trend of “up-down-up-down-up-down-up”. The risk level fluctuates between higher and general risks, with a downward trend overall. The RR system shows a steady downward trend. The risk level decreases from higher risk to general risk, and then decreases to lower risk.

### Spatial dimension analysis

**Figure 7:**
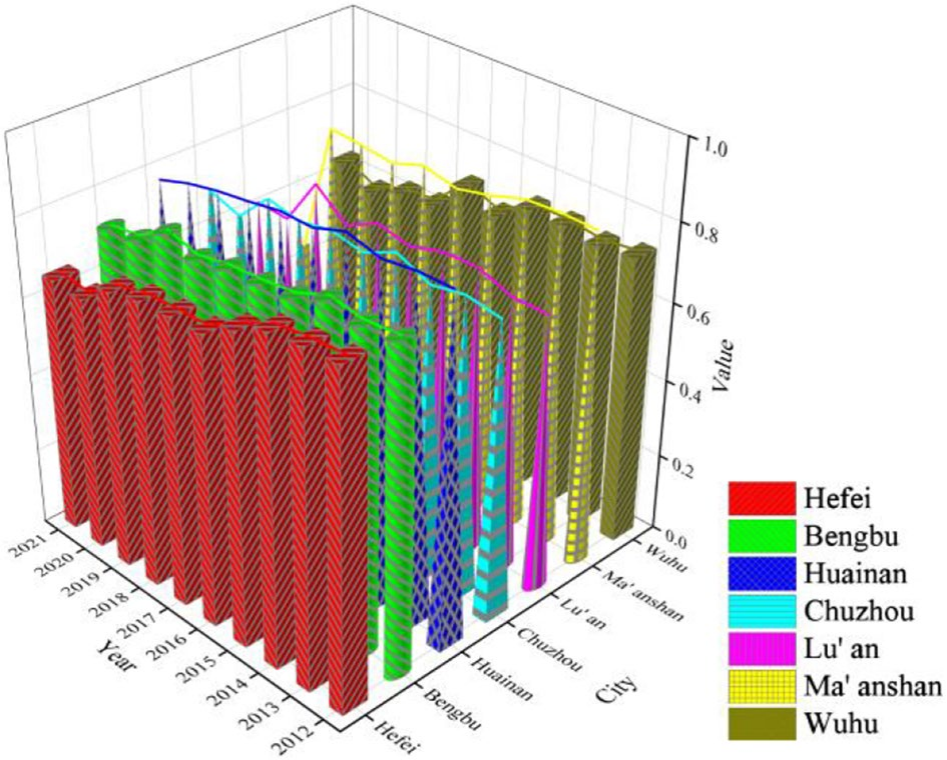
Three-dimensional histogram of ecological risk in soil and water resources.

In order to further explore the changes in the ecological risk of soil and water resources in the cities of Hefei metropolitan area, the ecological risk values of the seven cities in Hefei metropolitan area are compared from 2012 to 2021, as shown in Fig. 7. With the help of three-dimensional histogram, the changes in each city can be more intuitively seen.

The trend of ecological risk value changes in the past 10 years shows that Hefei City shows a steady decline, with a slight increase in 2021. Except for 2020 and 2021, when the risk grade was lowered to general and the warning grade was medium, the risk grade in the rest of the years was higher and the warning grade was heavy.

Bengbu City has an overall linear decline, with slight increases in 2014 and 2019. The overall risk grade remained at higher in all years, with a heavy warning grade.

Huainan City showed a steady downward trend, rising slightly and peaking in 2015, and fluctuating slightly upward in 2016–2017. In the first 6 years, the risk level is extremely high and the warning grade is huge, but in the last 4 years, the risk grade drops to higher and the warning grade is heavy.

Chuzhou City shows fluctuating changes in the first 8 years, reaching the highest value in 2015, plummeting to the lowest value in 2020, and recovering slightly in 2021. Except for 2020, when the risk grade is reduced to general and the warning grade is medium, the risk grade in all other years is higher and the warning grade is heavy.

Lu'an City is basically flat in the first 2 years, then increases and then decreases in 2013–2015, followed by a “W”-shaped trend, reaching the highest value in 2019 and dropping to the lowest value in 2020, with a slight rebound in 2021. The risk grade in 2014–2017 and 2019 is higher, and the warning grade is heavy. For the other years, the risk grade decreases to general, with a medium warning grade.

Ma'anshan City is essentially flat for the first 3 years, then shows slight fluctuating changes, but falls off a cliff in 2021. Except for 2021 when the risk grade drops to lower and the warning grade is light, the risk grade for the rest of the years is higher and the warning grade is heavy.

Wuhu City was basically flat in the first 4 years, followed by a "V"-shaped trend, and then a steady decline, reaching a minimum in 2020 and a slight recovery in 2021. In the first 4 years and 2017, the risk grade is higher and the warning grade is heavy. In the other years, the risk grade is lowered to general and the warning grade is medium.

**Figure 8:**
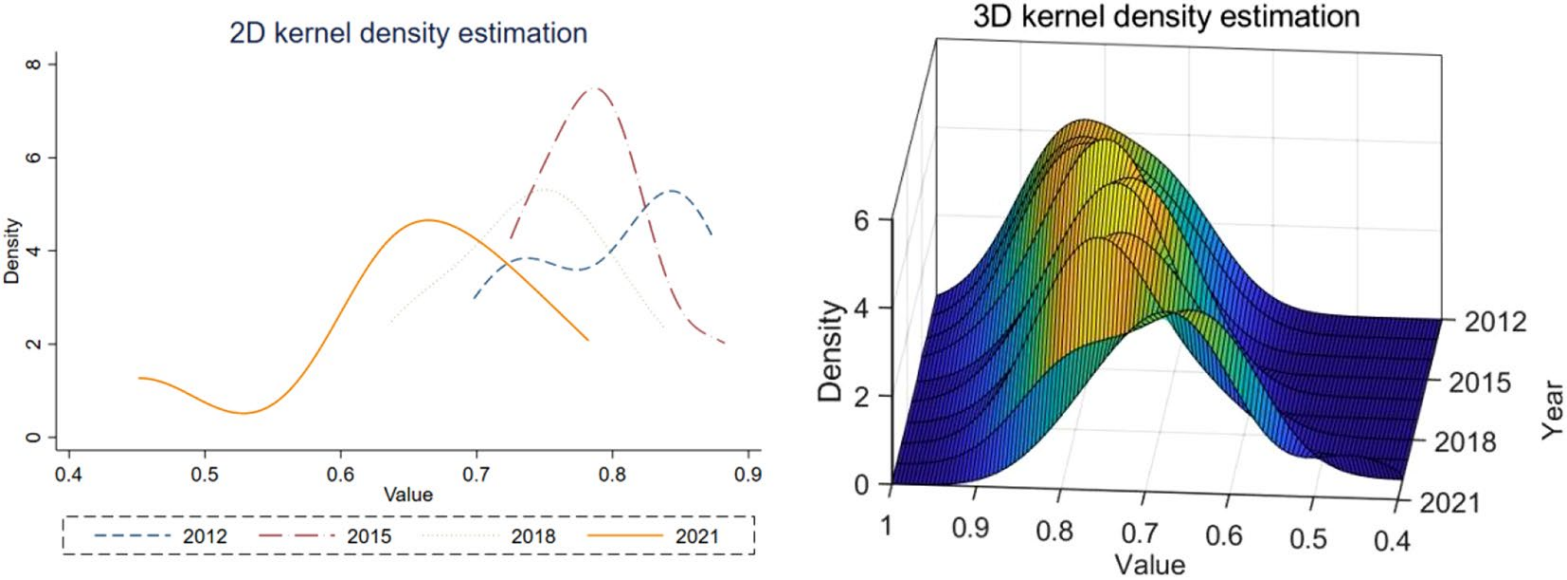
Kernel density map of ecological risk in soil and water resources.

With the help of Kernel density estimation to draw two-dimensional Kernel density map and three-dimensional Kernel density map, through the continuous density curve describes the distribution pattern of random variables, can be more intuitively analysed Hefei metropolitan area municipalities of the trend of dynamic change, as shown in Fig. 8.

From the kernel density map, the height of the distribution curve peaks firstly rises and then falls, and the width firstly narrows and then increases, indicating that the ecological risk difference of soil and water resources of each city in Hefei metropolitan area decreases first and then increases. The peak of the wave reaches the highest value in 2015, and the width of the wave reaches the smallest value, indicating that the cities of Hefei metropolitan area appear to have a good development trend of “progressing in tandem”. The wave peak gradually moves to the left, indicating that the ecological risk level of soil and water resources in the Hefei metropolitan area is declining. The phenomenon of trailing to the right in the first period and trailing to the left in the later period indicates that the ecological risk of soil and water resources in the Hefei metropolitan area still exists in the phenomenon of “high-value” agglomeration.

**Figure 9:**
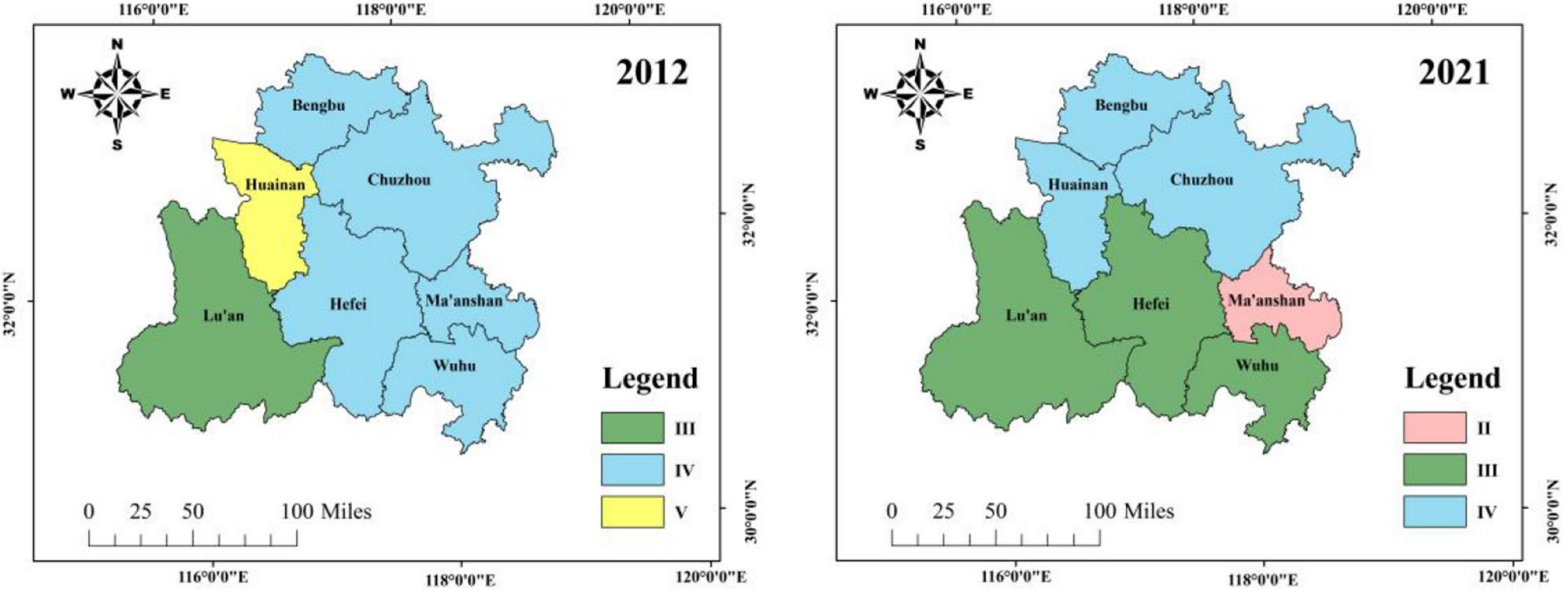
Geographical distribution changes of ecological risks of soil and water resources.

From the perspective of geographical distribution, as shown in Fig. 9.

The ecological risk level of soil and water resources in the Hefei metropolitan area in 2012 was generally concentrated at the higher risk level of Grade IV, with only two cities at the general risk level of Grade III and the extremely high risk level of Grade V. In 2021, the number of cities with ecological risk levels of Grade III and Grade IV was the same, with only Ma'anshan City falling to the lower risk level of Grade II. It can be seen that the ecological risk of soil and water resources in the Hefei metropolitan area is showing a positive development trend.

### Subsystem layer analysis

**Figure 10:**
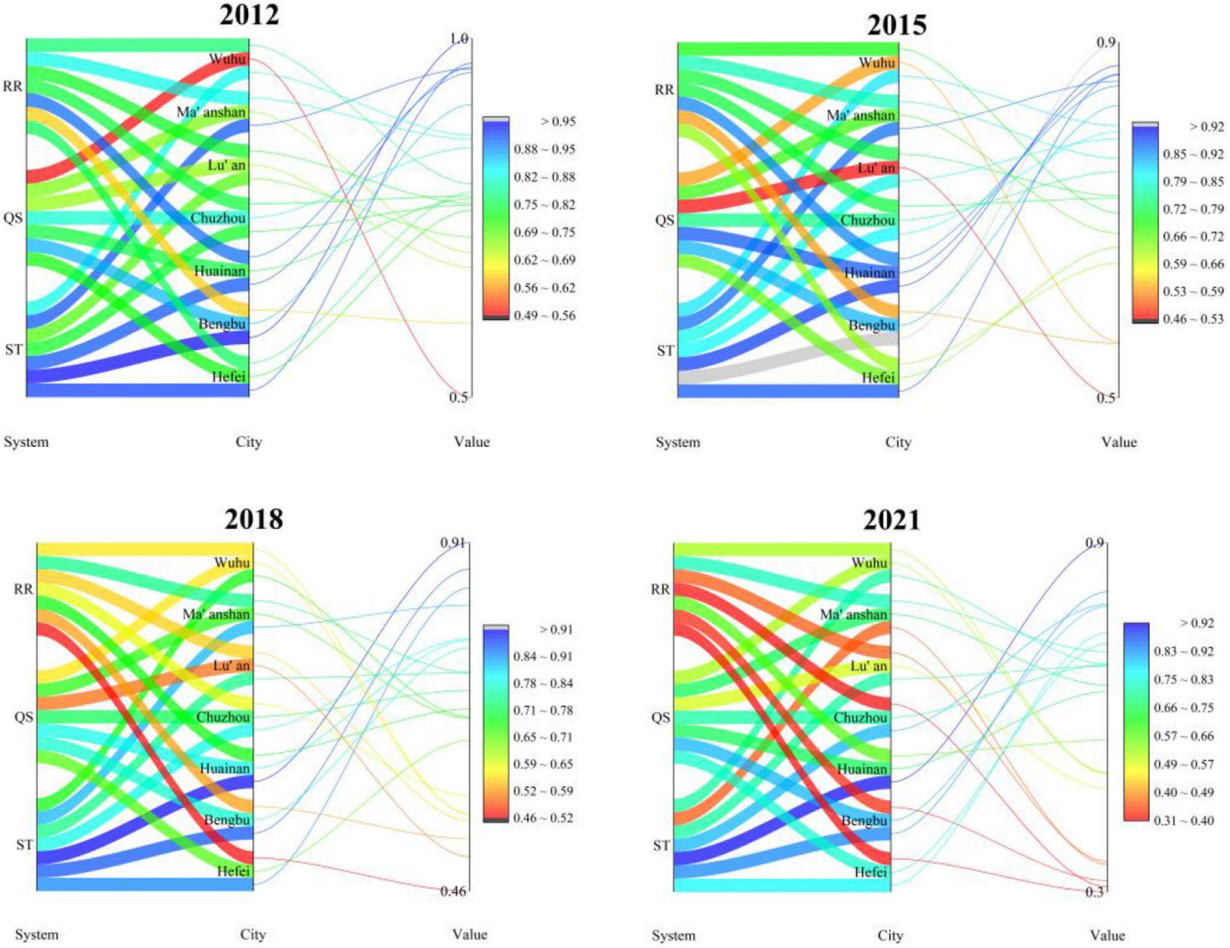
Plot of parallel coordinates of changes in the each municipal subsystems.

In order to deeply analyse the intrinsic evolution law of ecological risk of soil and water resources in Hefei metropolitan area, this paper explores it with the help of three subsystems, namely, security threat (ST), quality status (QS) and risk regulation (RR), and the trend of each subsystem is shown in Table 6 and Fig. 10.

**Table 6:** The changing trends of Hefei metropolitan area subsystems.

Subsystem/year	2012	2015	2018	2021
ST	0.850	0.838	0.807	0.730
QS	0.711	0.684	0.665	0.659
RR	0.759	0.705	0.586	0.453

The ST, QS and RR systems in the Hefei metropolitan area show a steady decline. the risk grade of the ST system is reduced from extremely high to higher, and the warning grade is lowered from huge to heavy. The risk grade of the QS system is reduced from higher to general, and the warning grade is lowered from heavy to medium. The risk grade of the RR system is reduced from higher to lower, and the warning grade is lowered from heavy to light.

Hefei City's ST and RR systems show a steady decline, while the QS system first declines and then rises. The risk grade of the ST system decreases from extremely high to higher, and the warning grade decreases from huge to heavy. The risk grade of the QS system first decreases from higher to general, and the warning grade decreases from heavy to medium, and then rises back to the original risk and warning grades. The risk grade of the RR system decreases from higher to lower, and the warning grade decreases from heavy to light.

The three subsystems in Bengbu City have the same trend as in Hefei City. The risk grade of both the ST and QS systems has been reduced from extremely high to higher, and the warning grade has been lowered from huge to heavy. The risk grade of the RR system has been reduced from general to lower, and the warning grade has been lowered from medium to light.

In Chuzhou City, the ST system shows a slanting “N” trend, while the QS system and RR system show a steady decline. The risk grade of the ST system and QS system has been kept at a higher, and the warning grade is heavy. The risk grade of the RR system has been reduced from a higher to lower, and the warning grade has been reduced from severe to light.

The ST and RR systems in Lu’an City have the same trend as Chuzhou City, with the QS system fluctuating and decreasing. The risk grade of the ST system has risen from general to higher, and the warning grade has risen from medium to heavy. The risk grade of the QS system has fluctuated between general and lower, and the warning grade has varied between medium and light. The risk grade of the RR system has decreased from higher to lower, and the warning grade has dropped from heavy to light.

Ma'anshan City's ST and RR systems show a steady decline, and the QS system is basically flat. The risk grade of the ST system has been reduced from extremely high to lower, and the warning grade has been lowered from huge to light. The risk grade of the QS system has remained at general, and the warning grade has been set at medium. The risk grade of the RR system has been maintained high, and the warning grade has been set at heavy.

Wuhu City's ST system declined and then increased, the QS system increased and then decreased, and the RR system showed a steady decline. The risk grade of the ST system was first reduced from higher to general, and the warning grade was lowered from heavy to medium, and then both were upgraded back to the original grade.The risk grade of the QS system was increased from lower to general, and the warning grade was raised from light to medium. The risk grade of the RR system was reduced from higher to general, and the warning grade was lowered from heavy to medium.

Comparing the ecological risk levels of water and soil resources in the seven urban subsystems of the Hefei metropolitan area, it can be seen that the urban subsystem levels generally show a downward trend, with some subsystems showing a slight upward trend. From the ST system, Hefei City, Bengbu City, and Ma'anshan City showed a steady downward trend. Huainan City, Chuzhou City, and Lu’an City showed a slight upward trend. From the QS system, Bengbu City, Huainan City, Chuzhou City, Lu'an City, and Ma’anshan City showed a downward trend. However, Hefei City and Wuhu City showed an upward trend. From the RR system, all cities in the Hefei metropolitan area showed a steady downward trend.

### Dynamic evolutionary analysis

With the help of traditional and spatial Markov chain to identify the dynamic transfer characteristics of the ecological risk of soil and water resources in Hefei metropolitan area and to explore its dynamic evolution pattern. The traditional Markov chain are shown in Table 7, and the spatial Markov chain are shown in Tables 8, 9 and 10.

**Table 7:** Traditional transfer probability matrix.

Number of intervals	Type of domain	L	ML	MH	H	Frequency
		< 25%	25% ~ 50%	50% ~ 75%	> 75%	
1	L	0.7692	0.2308	0.0000	0.0000	13
	ML	0.3125	0.4375	0.2500	0.0000	16
	MH	0.1176	0.2941	0.5882	0.0000	17
	H	0.0000	0.0000	0.2353	0.7647	17
3	L	0.7143	0.2857	0.0000	0.0000	7
	ML	0.6429	0.1429	0.2143	0.0000	14
	MH	0.1667	0.4167	0.4167	0.0000	12
	H	0.0000	0.1250	0.5625	0.3125	16
5	L	1.0000	0.0000	0.0000	0.0000	4
	ML	0.6000	0.2000	0.2000	0.0000	10
	MH	0.4286	0.4286	0.1429	0.0000	7
	H	0.0714	0.1429	0.5714	0.2143	14

**Table 8:** Matrix of spatial transfer probabilities. (T = 1).

Type of domain	T/T + 1	L	ML	MH	H	Frequency
		< 25%	25–50%	50–75%	> 75%	63
L	L	1.0000	0.0000	0.0000	0.0000	3
	ML	0.0000	1.0000	0.0000	0.0000	1
	MH	0.5000	0.0000	0.5000	0.0000	2
	H	0.0000	0.0000	0.0000	0.0000	0
ML	L	0.8000	0.2000	0.0000	0.0000	5
	ML	0.5000	0.2500	0.2500	0.0000	4
	MH	0.1667	0.1667	0.6667	0.0000	6
	H	0.0000	0.0000	0.0000	0.0000	0
MH	L	0.6667	0.3333	0.0000	0.0000	3
	ML	0.3750	0.5000	0.1250	0.0000	8
	MH	0.0000	0.3750	0.6250	0.0000	8
	H	0.0000	0.0000	0.2353	0.7647	17
H	L	0.5000	0.5000	0.0000	0.0000	2
	ML	0.0000	0.3333	0.6667	0.0000	3
	MH	0.0000	1.0000	0.0000	0.0000	1
	H	0.0000	0.0000	0.0000	0.0000	0

**Table 9:** Matrix of spatial transfer probabilities. (T = 3).

Type of domain	T/T + 1	L	ML	MH	H	Frequency
		< 25%	25–50%	50–75%	> 75%	49
L	L	0.0000	0.0000	0.0000	0.0000	0
	ML	0.0000	0.0000	0.0000	0.0000	0
	MH	0.0000	1.0000	0.0000	0.0000	1
	H	0.0000	0.0000	0.0000	0.0000	0
ML	L	1.0000	0.0000	0.0000	0.0000	2
	ML	1.0000	0.0000	0.0000	0.0000	4
	MH	0.3333	0.3333	0.3333	0.0000	3
	H	0.0000	0.0000	0.0000	0.0000	0
MH	L	0.6667	0.3333	0.0000	0.0000	3
	ML	0.5714	0.2857	0.1429	0.0000	7
	MH	0.1429	0.2857	0.5714	0.0000	7
	H	0.0000	0.1250	0.5625	0.3125	16
H	L	0.5000	0.5000	0.0000	0.0000	2
	ML	0.3333	0.0000	0.6667	0.0000	3
	MH	0.0000	1.0000	0.0000	0.0000	1
	H	0.0000	0.0000	0.0000	0.0000	0

**Table 10:** Matrix of spatial transfer probabilities. (T = 5).

Type of domain	T/T + 1	L	ML	MH	H	Frequency
		< 25%	25–50%	50–75%	> 75%	35
L	L	0.0000	0.0000	0.0000	0.0000	0
	ML	0.0000	0.0000	0.0000	0.0000	0
	MH	0.0000	0.0000	0.0000	0.0000	0
	H	0.0000	0.0000	0.0000	0.0000	0
ML	L	1.0000	0.0000	0.0000	0.0000	1
	ML	1.0000	0.0000	0.0000	0.0000	1
	MH	0.0000	1.0000	0.0000	0.0000	1
	H	0.0000	0.0000	0.0000	0.0000	0
MH	L	1.0000	0.0000	0.0000	0.0000	1
	ML	0.8333	0.1667	0.0000	0.0000	6
	MH	0.6000	0.2000	0.2000	0.0000	5
	H	0.0714	0.1429	0.5714	0.2143	14
H	L	1.0000	0.0000	0.0000	0.0000	2
	ML	0.0000	0.3333	0.6667	0.0000	3
	MH	0.0000	1.0000	0.0000	0.0000	1
	H	0.0000	0.0000	0.0000	0.0000	0

When the time span is 1 year, the probabilities on the main diagonal are all greater than those on the non-diagonal, and the probabilities that cities with ecological risk of soil and water resources of L, ML, MH, and H will still maintain their own status after 1 year are 76.92%, 43.75%, 58.82%, and 76.47%, respectively, which are large compared to the probabilities of transferring to a higher or lower risk, suggesting that the Hefei metropolitan area has a certain short-term inertia development trend, there is a more obvious Matthew effect, and at the same time there is a phenomenon of convergence of the four types of clubs. In addition, the stability of clubs located in the L and H risk types is significantly higher than that of clubs located in the ML and MH risk types, indicating that the path of ecological risk enhancement of soil and water resources is locked in a short period of time, and it is difficult to get rid of resource dependence.

When the time span is 3 and 5 years, the probabilit of cities continuing to maintain their status under the four types: 71.43% and 100.00% for L risk, 14.29% and 20.00% for ML risk, 41.67% and 14.29% for MH risk, and 31.25% and 21.43% for H risk. This shows that there is still a convergence of cities in the L risk type over a long time span. With the exception of the L risk type, the other risk types continue to decrease with increasing time spans, and the “club convergence” phenomenon is weakening.

In the time span of 1, 3 and 5 years, there is no transfer of other risk types to the H risk type. the H risk type initially transfers only to the MH risk type, to a trend of successive transfers to the ML risk and the L risk types. The cross-horizontal transfer probability of each city in the Hefei metropolitan area municipalities is smaller than the adjacent horizontal transfer probability, which is characterised by a certain inertia development.

Comparison of Tables 7 and 8 shows that there is a large difference in the transfer of municipal risk status after conditioning on different city soil and water resource risk types.

When the time span is 1 year, for cities with L and ML risk types, the higher the domain type, the higher the probability of upward transfer of its risk type when the city with a higher risk type compared to itself is used as a neighbour. And the higher the level of domain risk, the more sensitive it is. For cities with MH risk type, the probability of shifting downwards to ML type is higher as the domain type is higher. Cities with H risk type shift downwards successively.

Changes over time spans of 3 and 5 years have the same pattern of change, as shown in Tables 9 and 10.

### Resistance diagnostic analysis

According to formula (17) and combined with the weights obtained from the CRITIC method, the resistance values of the evaluation indicators and of the subsystems in 2021 were calculated. The top 4 resistance factors were ranked according to the resistance value from largest to smallest, as shown in Table 11.

**Table 11:** Ranking of major resistance factors.

City	Item	Factor 1	Factor 2	Factor 3	Factor 4
Hefei	Resistance value	0.166	0.153	0.147	0.119
	Indicator	I_3_	I_1_	I_12_	I_8_
Bengbu	Resistance value	0.127	0.110	0.106	0.091
	Indicator	I_5_	I_4_	I_9_	I_8_
Huainan	Resistance value	0.165	0.117	0.091	0.089
	Indicator	I_15_	I_5_	I_13_	I_1_
Chuzhou	Resistance value	0.156	0.116	0.101	0.089
	Indicator	I_9_	I_8_	I_5_	I_10_
Lu'an	Resistance value	0.158	0.141	0.141	0.110
	Indicator	I_6_	I_3_	I_13_	I_9_
Ma'anshan	Resistance value	0.178	0.133	0.126	0.117
	Indicator	I_15_	I_3_	I_8_	I_12_
Wuhu	Resistance value	0.189	0.146	0.108	0.095
	Indicator	I_12_	I_14_	I_1_	I_11_

**Figure 11:**
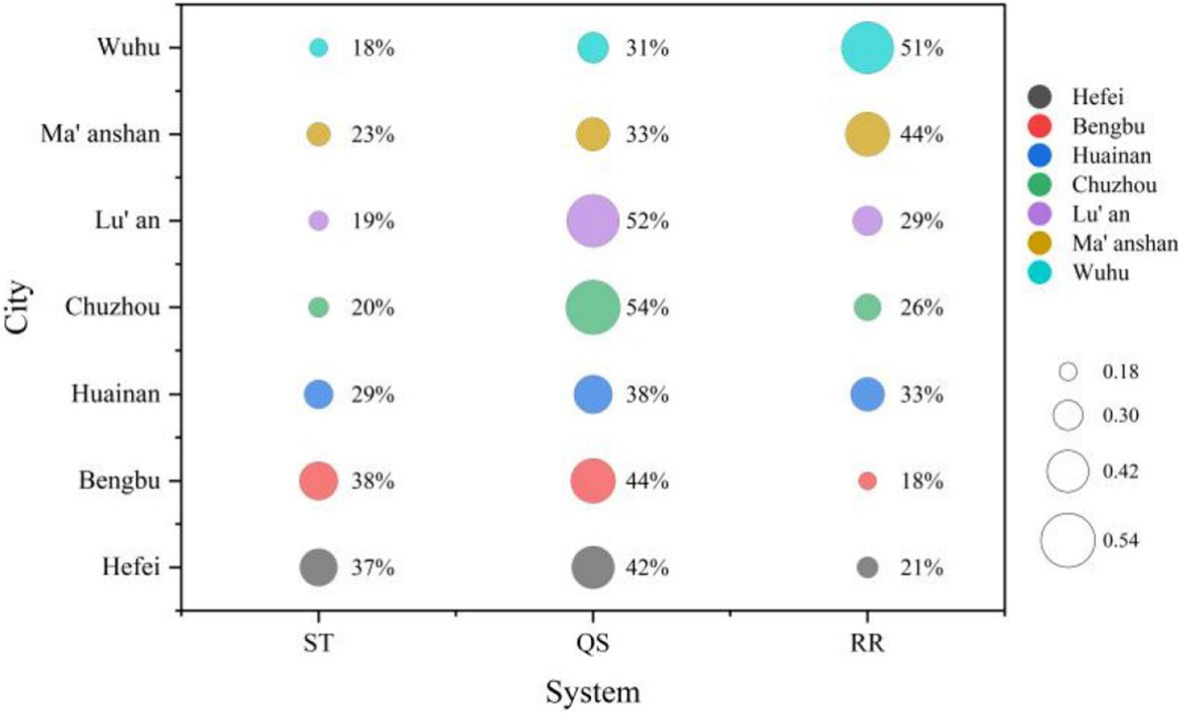
Bubble diagram of major resistance factors in 2021.

According to the resistance value of each municipal subsystem, it was plotted as a bubble diagram, as shown in Fig. 11.

Combining Table 11 and Fig. 10, it can be seen that the main subsystem constraining the Hefei city is the QS system (44%), which contains the proportion of green squares to construction land, etc. However, the first and second resistance factors are in the ST system (37%), which are urban population density and per capita water use for urban public services, respectively.

The resistance factors constraining Bengbu City are mainly concentrated in the QS system (44%), containing the matching coefficient of agricultural water and soil resources and the proportion of green squares to construction land. However, the first and second resistance factors are in the ST system (38%), which are the proportion of land resettlement and the proportion of groundwater extraction, respectively.The main subsystem constraining Huainan City is the QS system (38%), but the main resistance factors are in the RR system (33%) and the ST system (29%), which are the industrial wastewater treatment rate, per capita expenditure on research, the proportion of land resettlement, and per capita water use for urban public services, respectively.

The resistance factors constraining Chuzhou city were mainly concentrated in the QS system (54%), including the matching coefficient of agricultural water and soil resources, the proportion of green squares to construction land, and the per land rainfall. The resistance factors constraining Lu'an City are mainly concentrated in the QS system (52%), including urbanisation rate and matching coefficient of agricultural water and soil resources, etc. The ST system (19%) has the least resistance.

The resistance factors constraining Ma'anshan are mainly concentrated in RR systems (44%), including the industrial wastewater treatment rate and the proportion of public budget expenditure on agriculture, forestry and water. This is followed by the QS system (33%). The resistance factor constraining Wuhu City is mainly concentrated in the RR system (51%), including the proportion of public budget expenditure on agriculture, forestry and water and the Eco-environmental water supplement ratio. The ST system (18%) has the least resistance.

From the perspective of the resistance level of subsystems, the resistance factors that cause ecological risks in soil and water resources in various cities are mostly concentrated in the QS system. In addition to Ma'anshan and Wuhu, the resistance factors are concentrated in the RR system. From the perspective of the resistance level of major factors, I_8_ (Proportion of green squares to construction land) appears most frequently among the top four resistance factors. I_15_ (Industrial Wastewater Treatment Rate) appears most frequently among the top-ranked factors.

## Discussion

1. In terms of the feasibility of the study, the Hefei metropolitan area presents a spatial distribution pattern of “high in the north and low in the south, high in the west and low in the east” in the results of this study, which is in line with the viewpoints of Fan^26^, Wang^41^ and other scholars. This study provides a new theoretical perspective for the ecological risk evaluation of regional soil and water resources, and the research ideas and evaluation methods can be applied to the ecological risk evaluation of other regions. However, the study has the following shortcomings:

First, the years of the study data are not many, and the change of the multidimensional kernel density map is not too obvious, so it can be re-screened to construct a new evaluation indicator system by screening the evaluation indicators with greater time span that are highly obtainable and representative.

Second, the resistance model can only identify the resistance factors and fails to explore the driving factors, which can be combined with geodetectors.

Thirdly, the spatial weight matrix only uses proximity as a criterion, and elements such as geographical distance, economic differences and population density should be added.

(2) From the viewpoint of future research trends, the research on ecological risk of soil and water resources should have the following breakthroughs:

Firstly, arable land resources in land resources are facing the problem of spatial mismatch with water resources. How to optimise the spatial allocation of farmland soil and water resources and carry out the evaluation of the sustainable use of water resources in supplementary arable land has become a hot spot in today's research.

Secondly, for different geographic environments and based on different subject dimensions, the evaluation indicator system of "grading and zoning" should be constructed, and the coupling and coordination between each subject dimension should be strengthened. How to achieve the optimised cross-regional supply mode of "grading and zoning", and to strengthen the synergistic and optimal management of soil and water resources have also become the first priority of the construction of ecological civilisation.

Thirdly, the statistical data of soil and water resources must be timely, and an evaluation platform for geo-dynamic monitoring should be established. This platform should fulfil the functions of real-time processing of soil and water conservation information, comprehensive evaluation of soil and water conservation effects, dynamic prediction of soil and water erosion, and emergency management of soil and water erosion control.

## Conclusion

Based on the ST-QS-RR conceptual model, this paper scientifically selects 15 indicators to construct the evaluation system. The CRITIC-TOPSIS method is used to calculate the ecological risk value of soil and water resources in Hefei metropolitan area, and combined with the multi-dimensional kernel density method and spatial Markov chain to explore the evolution law and dynamic characteristics, and the resistance model is used to diagnose the main resistance factors and resistance subsystems. The results can reflect the ecological risk level of soil and water resources of each city in Hefei metropolitan area and the evolution trend of the risk warning, and the specific conclusions are as follows.

1. Analysed from the time series and spatial dimension, the ecological risk of soil and water resources in the Hefei metropolitan area is relatively stable, with a steadily declining trend. The ecological risk difference of soil and water resources in each city decreased first and then increased, showing a downward trend as a whole and the spatial distribution pattern of “high in the north and low in the south, high in the west and low in the east”, with the phenomenon of “high value” agglomeration.
2. Analysed from the subsystem layer and resistance diagnosis, the subsystems in the Hefei metropolitan area all show a steady downward trend. Most of the subsystems in the cities show a decreasing trend, and individual subsystems are slightly increasing. Most of the resistance factors that cause ecological risk of soil and water resources in each city are concentrated in the QS system, and the main resistance factor I_8_ (the proportion of green squares to construction land) has the highest frequency.
3. From the evolution law and dynamic characteristics analysis, there is no transfer of other risk types to H risk type in Hefei metropolitan area, and the probability of cross-horizontal transfer is smaller than the probability of adjacent horizontal transfer, with inertial development characteristics and the existence of club convergence phenomenon. When the adjacent domain type is higher, the probability of upward transfer of risk type is higher, and the risk level change is more sensitive.

Based on the above conclusions, this article proposes the following countermeasures for the ecological risks of soil and water resources in the Hefei metropolitan area:

1. Comprehensively strengthen the prevention and protection of soil erosion. Emphasis on the prevention and control of soil erosion at its source, and implement differentiated protection and management measures. Strengthen the prevention and protection of key areas, and promote the planning of ecological restoration of land space in Anhui Province. Improve the soil and water conservation function of ecosystems, and promote urban soil and water conservation and ecological restoration.
2. Strictly supervise the human-induced soil erosion according to the law. Improve the supervision system and standards, and perfect the standards for the prevention and control of soil erosion in agricultural and forestry development and other production and construction activities. Innovate and improve the supervision methods, and carry out the remote sensing supervision of soil and water conservation in a full coverage and normalized way. Strengthen the implementation of corporate responsibility, strictly implement the “three simultaneous” requirements for soil and water conservation.
3. Improve the management ability and level of soil and water conservation. Improve the planning system of soil and water conservation, strengthen the tracking, monitoring and evaluation of the implementation of the plan. Strengthen the monitoring and evaluation of soil and water conservation, optimize the layout of soil and water conservation monitoring stations. Strengthen the scientific and technological innovation of soil and water conservation, strengthen basic research and key technology research.

### Supplementary Information

Supplementary Information.

## Data Availability

For Fig. 1, 2, 3, 4, 5 and 9, all map data in this article are sourced from https://www.resdc.cn/Default.aspx, the ArcMap download website for drawing maps is https://desktop.arcgis.com/zh-cn/desktop/index.html. All data used for this study could be made available on request with corresponding author.
